# Case Report: COVID Associated Pancytopenia Unmasking Previously Undiagnosed Pernicious Anemia

**DOI:** 10.4269/ajtmh.21-1194

**Published:** 2022-04-11

**Authors:** Javier J. Barranco-Trabi, Robert Minns, Roushon Akter, Katherine Park, Sean Babb, Jennifer Masel

**Affiliations:** ^1^ Department of Internal Medicine, Tripler Army Medical Center, Honolulu, Hawaii;; ^2^ Infectious Disease-Service, Tripler Army Medical Center, Honolulu, Hawaii

## Abstract

Severe acute respiratory syndrome coronavirus 2 (SARS-CoV-2)-associated pancytopenia is a known but rare complication of COVID-19 syndrome that is not well described in literature. Severe acute respiratory syndrome coronavirus 2 has shown the potential to affect any organ including the bone marrow, which then results in a decrease in all three blood cell lines. These cases usually resolve with the passage of time and treatment of underlying risk factors. As COVID pneumonia rates continue to increase worldwide, it is crucial to be able to recognize this complication. Additionally, deeper investigation into patient’s response to COVID infection can be complicated by unexpected underlying disease. We report a case of a symptomatic 24-year-old active duty male in Hawaii with post-COVID pancytopenia that was found to have previously undiagnosed pernicious anemia and his response to standard treatment.

## INTRODUCTION

COVID-19 syndrome is caused by the severe acute respiratory syndrome coronavirus 2 (SARS-CoV-2). Generally, those affected with COVID-19 manifest with respiratory pathology, especially the classic bilateral multifocal pneumonia; less commonly, however, the infection affects the bone marrow, hindering the hematopoietic system.^1^ This may lead to lymphopenia, neutropenia, and rarely pancytopenia.^2^^,^^3^ The first case of persistent pancytopenia associated with SARS-CoV-2 bone marrow infiltration in an immunocompromised patient was described in October 2020, and subsequently other reports emerged.^4^^,^^5^

Our purpose is to present clinical findings of a rare case of an immunocompetent young individual with COVID-19-associated pancytopenia with masked pernicious anemia. Although our case shares some similarities with cases reported previously, there are also differences that merit consideration.

## CASE PRESENTATION

A 24-year-old active duty African American male presented to emergency room 18 days post COVID pneumonia infection, complaining of persistent fatigue, weakness, and subjective weight loss. He stated that his initial symptoms of dysgeusia, anosmia, headache, and fever had previously resolved since his initial COVID-positive test. He had no family history of blood disorders and had no significant past medical and surgical history. He was on no medications or supplements at the time of admission. Initial investigation with a complete blood count revealed a hemoglobin (HGB) of 4.1 g/dL, white blood cells (WBC) of 4.1 g/dL, and platelets (PLTs) of 64 × 10(9)/L. His anemia was normocytic (mean corpuscular volume [MCV] 95.3) and normochromic (mean corpuscular hemoglobin [MCH] 32.0). His leukopenia was lymphocytes predominant (55% lymphocytes, 33% granulocytes, and 6% monocytes). A peripheral smear was done which showed moderate oval macrocytes, anisocytosis, poikilocytosis, schistocytes, and atypical lymphocytes most consistent with a hypocellular sample with dilution. The patient was admitted to the medicine ward for blood transfusion, volume resuscitation, and further workup.

Diagnostic evaluation for hypo-proliferation, hemolysis, and malnutrition was initiated. There was concern for a hemolytic process so a bilirubin level was obtained, which was elevated at 3.4 mg/dL with indirect bilirubin predominance at 1.8mg/dL, and lactate dehydrogenase was elevated suggesting a hemolytic anemia. However, his reticulocyte index was 0.13, consistent with hypo-proliferation. Direct antithrombin test was also negative. He was transfused 2 units of pack red blood cells and had an appropriate rise in hemoglobin to > 7 g/dL. We continued to work the patient up with multiple viral panels, *Leptospirosis*, cold agglutinins, erythrocyte sedimentation rate (ESR), and antinuclear antibodies (ANA) to rule out other causes of his presentation. Flow cytometry cytogenetic analysis revealed no evidence of an acquired clonal abnormality.

**Figure 1.**
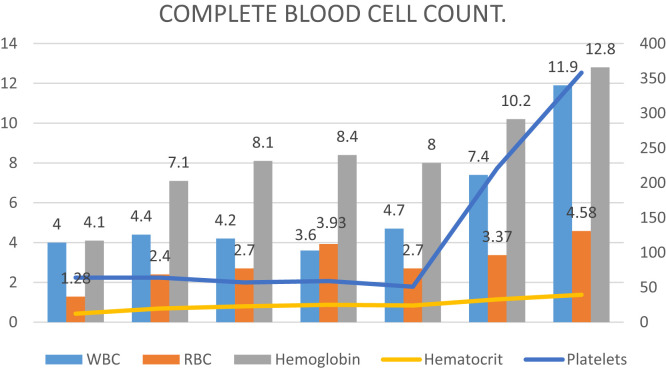
Complete blood cell count. Values in this table were taken from the admission until a month after discharge. This figure appears in color at www.ajtmh.org.

A bone marrow biopsy was performed to exclude an underlying malignancy given the very high LDH. The biopsy showed no evidence of an increased blast population, no immunophenotypic evidence of a B-cell or a T-cell lymphoproliferative disorder and no immunophenotypic evidence of a plasma cell neoplasm. He continued to improve clinically with supportive care despite the uncertain pathology of his pancytopenia. His HGB, WBC, and PLTs continued to uptrend over the next month (see Figure 1). On the last day of admission, his B12 level was checked and found to be < 109 pg/mL (below reference range), with both homocysteine and methylmalonic acid elevated. The patient was started on B12 shots and discharged home with close follow-up. On further workup outpatient he was discovered to have parietal cell antibodies confirming a pernicious anemia.

## DISCUSSION

The COVID associated pancytopenia is a known but rare complication of the COVID-19 syndrome and usually presents in those with underlying risk factors such as diabetes, or prior malignancy.^4^^–^^7^ This was supported by our patient, where he was found to have a previously undiagnosed risk factor, pernicious anemia. Our patient was asymptomatic before his COVID infection and had no known risk factors which would have indicated a B12 deficiency. Although there are reports in literature of patients developing autoimmune disease post COVID infection, it is possible that our patient developed the B12 deficiency in the setting of a COVID response, although unlikely. Although a causal link between autoimmune disease and COVID has not been identified, it is suggested by temporal association.^8^

It is hypothesized that COVID causes myelosuppression by autoantibody targeted destruction of blood cells.^6^ Severe acute respiratory syndrome coronavirus 2 targets angiotensin converting enzyme 2 receptors, which have been also found in bone marrow.^6^ Thus, binding to these receptors can cause downstream effects including pancytopenia. Furthermore, there are case reports that have hypothesized that proinflammatory cytokines can impair hematopoiesis.^6^ With infection of SARS-CoV-2 in the lungs, the destruction of lung hematopoietic progenitors can result in pancytopenia.

The COVID pancytopenia is usually self-limited and will resolve with supportive care and addressing any underlying risk factors.^4^^–^^7^ Our case supports this, as seen by his improvement over admission with supportive care, and further return to normal cell line function on follow-up. The COVID pancytopenia, although rare, is still possible and should be on the differential for patients with recent COVID infections presenting with decrease of all three cells lines. Workup for concurrent causes should also be done, however, a bone marrow biopsy may not be necessary in the setting of improving pancytopenia with supportive care.^7^ We encourage clinicians to consider this before doing invasive tests.

## CONCLUSION

We are still in the process of investigating hematological alterations because of COVID-19.^2^ In the setting of COVID-induced pancytopenia it is important to cast a broad net of differentials to catch other risk factors. More studies are needed to determine whether this new virus presents notable differences in the mechanisms that produce pancytopenia with respect to others. But luckily, it appears that most patients with COVID-induced pancytopenia will recover marrow function as they recover from the COVID-19 syndrome with addressing of underlying risk factors.
